# Gene Therapy and Diabetes: A Narrative Review of Recent Advances and the Role of Multidisciplinary Healthcare Teams

**DOI:** 10.3390/genes16010107

**Published:** 2025-01-20

**Authors:** Nadia Khartabil, Ani Avoundjian

**Affiliations:** School of Pharmacy, Center of Graduate Studies, West Coast University, 590 N Vermont Ave, Los Angeles, CA 90004, USA; aavoundjian@westcoastuniversity.edu

**Keywords:** gene therapy, type 1 diabetes, type 2 diabetes, glycemic control, wound healing, VEFG (vascular endothelia growth factor), HGF (hepatocyte growth factor)

## Abstract

**Introduction:** Gene therapy has emerged as a promising frontier in the management of diabetes, offering innovative approaches to address both type 1 and type 2 diabetes. This narrative review examines the advancements in gene therapy applications, focusing on both animal and human studies, and includes a total of 11 studies in adherence to PRISMA guidelines. These studies utilize various viral vectors, such as adeno-associated virus (AAV) and lentivirus, to deliver genes that regulate insulin production and enhance angiogenesis. This review aims to synthesize recent advancements in gene therapy for both type 1 and type 2 diabetes and its complications, and to explore the evolving role of pharmacists in this emerging field. **Methods:** A comprehensive search was conducted to identify relevant studies on gene therapy for diabetes. Databases such as PubMed, the Cochrane Database of Systematic Reviews, the Cochrane Central Register of Controlled Trials, and Google Scholar were queried using keywords such as “Diabetes”, “gene therapy”, “Type 1 diabetes”, and “Type 2 diabetes”. Both animal and human studies were included to provide a broad perspective on the advancements in this field. **Results:** Animal model studies have shown promising results, including sustained insulin production, improved glucose homeostasis, and enhanced wound healing. Human studies, though fewer in number, have reported significant advancements. Patients with diabetic neuropathy treated with plasmid VEGF and recombinant adeno-associated virus (rAAV) showed improvements in neuropathic symptoms and glycemic control. Other studies involving intramuscular injections of VM202 and bicistronic VEGF165/HGF plasmid have reported pain reduction, improved healing of ischemic lesions, and increased angiogenesis. **Conclusions:** Despite these encouraging results, limitations such as small sample sizes, short follow-up periods, and the necessity for more extensive clinical trials persist. Diabetes is a metabolic syndrome that requires the collaboration of a multidisciplinary team to assist in several aspects of implementing successful gene therapy. Several healthcare providers and policy makers may play a crucial role in patient education, counseling, and the management of gene therapy treatments.

## 1. Introduction

### 1.1. Background and Significance

Diabetes mellitus is a complex, multi-pathway chronic metabolic condition that can be caused by resistance to insulin, insulin-deficiency, autoimmune diseases, dysfunctional pancreatic function, elevated glucose levels, lipids, oxidative stress, and more factors which are still to be determined [1]. Diabetes mellitus is classified into type 1 diabetes and type 2 diabetes, with both types resulting in elevated serum glucose levels. Long-term hyperglycemia inevitably leads to microvascular and macrovascular damage leading to organ dysfunction and/or failure, such as neuropathy, nephropathy, retinopathy, peripheral vascular disease, morbidity, and mortality. The prevalence of diabetes mellitus is increasing rapidly; there is a massive estimated diabetic population in the United States, with 38.4 million Americans diagnosed with type 1 or type 2 diabetes, the most prevalent category being type 2 diabetes mellitus. [1].

### 1.2. Current Therapeutic Approaches and Their Limitations

Current T1DM treatment focuses on the supplementation of insulin. Within T2DM therapeutic recommendations, several oral and injectable hypoglycemic agents have been approved. The American Diabetes Association (ADA) guidelines include medications such as metformin, sodium–glucose cotransporter-2 inhibitors (SGLT2 i), glucagon-like peptide receptor agonists (GLP-1 RAs), dipeptidyl peptidase-4 inhibitors (DPP-4 i), thiazolidinediones, and sulfonylureas [2]. All of the mentioned pharmacological agents have various glucose-lowering potentials, yet such interventions have not been able to control diabetes fully [3]. The limitations of these agents are the fact that while few agents ameliorate insulin sensitivity, none have demonstrated the ability to stop insulin resistance or a decrease in pancreatic function. With the evolution of gene therapy, the strategy of over- or under-expressing a genetic factor may have an impact on potentially more effective treatments or cures.

### 1.3. Gene Therapy: An Overview

The FDA defines gene therapy as a technique that modifies a person’s genes to treat or cure disease [4]. They can work by several mechanisms, such as replacing a disease-causing gene with a healthy copy of the gene, inactivating a disease-causing gene that is not functioning properly, and introducing a new or modified gene into the body to help treat a disease [5]. There is an abundant mode of delivery for gene therapy and an ever-increasing capability to do so [6].

### 1.4. Gene Therapy for Diabetes

Gene therapy for type 1 diabetes (T1D) has predominantly aimed at restoring insulin production by islet cells or their surrogates, or preventing the destruction of β cells. Conversely, gene therapy for type 2 diabetes (T2D) targets multiple mechanisms to enhance glucose tolerance, reduce insulin resistance, and improve cellular energy expenditure [7]. Gene therapy strategies can be categorized in several ways. One approach distinguishes between ex vivo therapy, where genes are modified in vitro before being introduced into the patient, and in vivo therapy, where genetic modifications occur directly within the patient. Another categorization is based on the delivery method, which can involve either viral or non-viral vectors.

Viral vectors are considered viruses which have a natural ability to deliver genetic material into cells [8]. Bacterial vectors can also be modified to prevent them from causing infectious disease, and can also be used as vectors themselves (otherwise known as vehicles) to carry therapeutic genes into human cells. Non-viral vectors are liposomes and other nanoparticles that deliver DNA or RNA to specific targeted cells which have lower immunogenicity. Stem cell-based gene therapy induces pluripotent stem cells, or iPSCs, which can then be genetically modified and differentiated into insulin-producing beta cells. Immune modulation is used to enhance the expression of FOXP3 in Tregs and increase IL-10 production, protecting the beta cells from autoimmune attacks [9]. Although a comprehensive discussion of these delivery methods and their advancements is beyond the scope of this paper, key advancements will be highlighted through the selected trials reviewed here.

### 1.5. Selected Gene Targets in Diabetes

Genes and gene expressions, as previously mentioned, have been targeted in several studies. For example, forkhead box P3, or FOXP3, deficiency and mutations can result in immune dysregulation and type 1 diabetes. An approach taken by gene therapy is to enhance FOXP3, which increases the number and function of Tregs, which are regulatory T-cells and a subset of CD4+, thereby promoting immune tolerance and protecting beta cells. Correcting FOXP3 mutations prevents type 1 diabetes if caught early, and will be a necessary tool in the future of gene therapy and its targets [10].

Glucokinase, or GCK, is a glucose sensor in beta cells and hepatocytes that aids in the body’s glucose homeostasis [11]. GCK converts glucose to glucose-6-phosphate, initiating the steps in glycolysis and helping regulate insulin secretion in response to blood sugar levels. Mutations in this gene can cause maturity-onset diabetes of the young, also known as MODY [12]. IL-10, an anti-inflammatory cytokine that is necessary for immune regulation, also suppresses inflammatory responses and protects against autoimmune responses that destroy beta cells in type 1 diabetes [13]. Having an altered or reduced IL-10 level contributes to chronic inflammation and beta cell destruction. Using gene transfer, viral vectors deliver the IL-10 gene to select tissues and enhance local anti-inflammatory effects which alleviate diabetic symptoms [14].

PDX1 is a transcription factor which is part of the development of the pancreas and the function of the pancreatic beta cells which produce insulin. Those deficient in PDX1 expression have impaired beta-cell function and insulin production, which inevitably leads to type 1 and type 2 diabetes. A gene therapy approach to restore this function is introducing functional PDX1 genes into beta cells to restore insulin production. The PDX1 is used to differentiate stem cells into insulin-producing beta cells. The approach to editing this gene by correcting the PDX1 mutation using CRISPR-Cas9 can activate or increase the activation of the gene, leading to the body’s restoration of insulin production. NK6 Homeobox 1, a transcription factor involved in the maintenance of beta cell identity and insulin secretion, causes the proliferation and survival of beta cells in the pancreas that improves their function and insulin production [15].

This review will look at the findings of gene therapy techniques performed on patients including viral vectors, non-viral vectors, stem cell-based gene therapy, immune modulation, and gene editing technologies such as CRISPR-Cas9, TALENs, zinc-finger nucleases, and more.

### 1.6. Rational and Objective of the Review

Existing research has primarily focused on the basic science and clinical trial outcomes of gene therapy for diabetes. However, there is a gap in the literature concerning the practical application of these therapies, with a summary of latest research findings needed to equip the pharmacist with the necessary knowledge to expand and bridge the continued care for gene therapy patients. This review aims to synthesize recent advancements in gene therapy for both type 1 and type 2 diabetes and its complications and to explore the evolving role of pharmacists in this emerging field.

## 2. Materials and Methods

### 2.1. Eligibility Criteria

A narrative review approach was chosen due to the diverse outcomes reported in the literature, which necessitated a flexible framework for exploring and interpreting the findings across varied study designs and contexts. This review followed the PRISMA 2020 guidelines [16]. Using PRISMA in this context ensures a structured and replicable methodology, providing clarity about the processes while accommodating the complexity of the topic. Inclusion criteria were as follows: the study type only included primary research studies such as randomized controlled trials (RCTs), cohort studies, case–control studies, and case series that investigate the application of gene therapy in the context of diabetes. The population included human participants diagnosed with any form of diabetes (type 1, type 2, gestational, etc.) or animal models closely related to diabetes (e.g., mice, rats). The intervention/exposure was gene therapy used as a treatment modality for diabetes, including but not limited to gene editing, gene transfer, or gene modulation techniques aimed at managing, preventing, or understanding the pathophysiology of diabetes. The outcome measures reported were relevant to diabetes, such as glycemic control, insulin sensitivity, pancreatic beta-cell function, diabetic complications, or other relevant clinical, biochemical, or molecular outcomes. The publication language was not restricted, provided that a translation could be obtained if necessary.

The exclusion criteria were any irrelevant studies that do not investigate gene therapy in relation to diabetes. Publications such as reviews, commentaries, and editorials were excluded. Animal studies unrelated to diabetes were also excluded. Studies with inadequate data or where the methodology is not clearly described were also excluded.

### 2.2. Information Sources

The databases searched consisted of PubMed, the Cochrane Database of Systematic Reviews, the Cochrane Central Register of Controlled Trials, and Google Scholar.

### 2.3. Search Strategy

The search strategy comprised searching through databases that included randomized controlled trials of all studies related to gene therapy AND diabetes. Several criteria were also looked at, including complications due to diabetes mellitus, diabetic retinopathy, diabetic neuropathy, and peripheral neuropathy as well as gene therapy delivery in mice, humans, canines, and rats.

### 2.4. Selection Process

The keywords and Boolean terms used included “diabetes”, “gene therapy”, “CRISPR-Cas9”, “type 1 diabetes”, “type 2” diabetes, “and”, “or”, “animal studies”, “clinical studies”, “randomized controlled trials”. All included studies were added to the Rayyan AI platform [17].

### 2.5. Data Collection Process

Authors were blinded to each other’s selections during the initial screening. They independently reviewed the articles for inclusion. After the initial selection, the authors met in two separate meetings to discuss and resolve any discrepancies in their decisions. Discrepancies were resolved through discussion, and if consensus could not be reached, a third author acted as an arbitrator.

### 2.6. Data Items

Data were extracted using a standardized form, capturing information on study design, population, interventions, outcomes, and key findings. The data extraction process was carried out independently by two authors, NK and GS, with discrepancies resolved through discussion.

### 2.7. Study Risk of Bias Assessment

The risk of bias assessment for the included studies was conducted using the mixed methods appraisal tool [18]. MMAT evaluates the methodological quality of studies based on specific criteria tailored to the study design. This includes qualitative studies, quantitative randomized controlled trials, quantitative non-randomized studies, quantitative descriptive studies, and mixed methods studies. For all types of studies, the initial screening questions remain consistent, ensuring a standardized preliminary evaluation. However, MMAT is not suitable for assessing studies involving animal models. Consequently, for studies involving animal models, we employed the Consolidated Standards of Reporting Trials (CONSORT) extension to appraise the quality of the research. The combination of MMAT and CONSORT extension was used by two authors, NK and GS, to assess the methodological quality and potential biases within the diverse study designs included in this review [19].

## 3. Results

### 3.1. Study Selection

**Figure 1:**
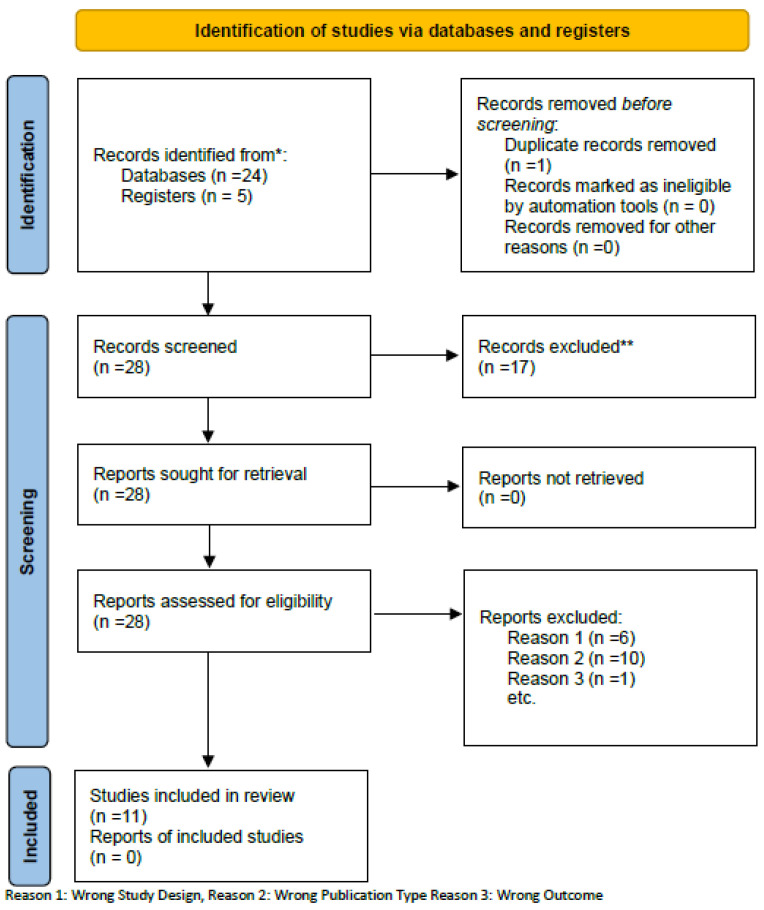
PRISMA 2020 flow diagram [16].

The initial search yielded a total of 29 articles. After removing duplicates (1), there were 28 articles for title and abstract screening. After screening, there were a total of 17 excluded articles. A total of 11 articles were included. These studies were selected for their relevance. The details of the PRISMA flow diagram can be reviewed in Figure 1.

### 3.2. Study Characteristics

The included studies listed two studies with T1D and T2D patients, three studies for diabetic complications treatment, and six animal studies. The follow-up period for human studies ranged from 3 months up to 5 years.

### 3.3. Risk of Bias Studies

The MMAT evaluation focused on clear research questions and the adequacy of collected data to address these questions. The criteria included the use of robust randomization protocols, similarity in baseline characteristics, the completeness of outcome data, the blinding of researchers, and the adherence of participants. Most human studies demonstrated high methodological quality, with randomized controlled trials employing double-blind designs and comprehensive data reporting. However, some studies showed limitations in participant adherence and incomplete data collection.

For animal studies, the CONSORT checklist was used to appraise methodological quality. The criteria included detailed reporting of titles and abstracts, backgrounds and objectives, methods, participant characteristics, interventions, outcomes, sample sizes, randomization, blinding, statistical methods, results, participant flow, recruitment, baseline data, numbers analyzed, outcomes and estimations, ancillary analyses, harms, discussion, limitations, generalizability, interpretation, registration, protocol, and funding. The detailed checklist is available in Table A1 in the Appendix A. Studies such as those by Kojima et al. and Elsner et al. showed thorough reporting and adherence to methodological rigor, with significant findings on blood glucose and insulin levels, as well as histological analysis which confirmed islet neogenesis and insulin expression [20,21]. However, the limitations included the lack of long-term follow-up and the generalizability of animal model results to human populations.

### 3.4. Results of Individual Studies

#### 3.4.1. Animal Studies

According to the outcomes of the animal studies, each study demonstrates that gene therapy can increase insulin production, maintain stability, and decrease complications caused by diabetes mellitus. Callejas’ results express the effectiveness of combined insulin and glucokinase gene therapy, with sustained glycemic control and stability for over 4 years [11]. Saaristo’s study utilized VEGF-C gene therapy to exhibit its impact on angiogenesis and lymph angiogenesis, accelerating wound healing by about 20% [22]. Sapir’s study had resulted in significant activation of insulin production in liver cells via PDX-1 induced trans-differentiation, lowering blood glucose levels [23]. Kojima’s study induced islet neogenesis in the liver using NeuroD and betacellulin, effectively restoring endogenous insulin production in mice [24]. Elsner’s results showcase the normalization of blood glucose levels in diabetic participants for over a year. Using lentiviral transduction to achieve hepatic insulin expression, Elsner was able to essentially cure diabetes mellitus. Handorf also used insulin gene therapy to demonstrate the normalization of blood glucose and increasing insulin levels [24].

#### 3.4.2. Human Studies

The human studies summarized here investigated the significance of gene therapy for improving glycemic control and addressing diabetes complications.

##### Glycemic Control, Insulin Production and Diabetic Complication Treatment

Hsu, P. Y. J. et al. analyzed type 1 and type 2 diabetes patients for five years in a controlled trial using a recombinant adeno-associated virus (rAAV) to deliver the human insulin gene. They reported enhanced glucose control and insulin production [25].

Barc et al. studied type 2 diabetes patients with critical limb ischemia, using an intramuscular injection of pIRES/VEGF165/HGF bicistronic plasmid (plasmid internal ribosome entry site/vascular endothelial growth factor 165/hepatocyte growth factor). In three months, they noted a statistically significant increase in serum VEGF levels, improvement in the ankle–brachial index (ABI), decreased rest pain, and improved vascularization [25]. VEGF (vascular endothelial growth factor) is a signal protein that stimulates the formation of blood vessels. HGF (hepatocyte growth factor) plays a crucial role in cell growth, cell motility, and angiogenesis. Kesler et al. conducted a phase III randomized controlled trial on patients with painful diabetic peripheral neuropathy, showing significant pain reduction and potential disease-modifying effects with the intramuscular injection of VM202 (a proprietary gene therapy product that is a non-viral, DNA-based treatment) over nine to twelve months. VM202 is a plasmid DNA that expresses two isoforms of HGF, designed to promote angiogenesis and tissue repair [26]. Ropper et al. conducted a controlled trial with 50 type 1 and type 2 diabetes patients over six months, using a plasmid VEGF delivery method. The study showed an improvement in diabetic neuropathic symptoms and pain reduction [27]. Kupczynska et al. focused on diabetic foot syndrome patients in a randomized controlled trial using an intramuscular injection of a bicistronic VEGF165/HGF plasmid. Over six months, they observed improved healing of ischemic lesions and increased angiogenesis. These studies collectively underscore the promise of gene therapy in treating both type 1 and type 2 diabetes, offering improved glycemic control, enhanced insulin production, and significant mitigation of diabetes-related complications [28]. A summary of the studies is listed in Table 1.

## 4. Discussion

### 4.1. General Interpretation of Animal Studies

The current animal studies on gene therapy for diabetes provide foundational insights and provide the proof-of-concept for the potential to translate these findings into clinical applications for human patients. For instance, Kojima, H. et al. demonstrated improved islet neogenesis and insulin production in diabetic mice using a lentivirus delivery method targeting NeuroD1 and Betacellulin, suggesting a viable approach for enhancing endogenous insulin production [16]. Elsner, M. et al. observed stable hepatic insulin expression and sustained blood glucose levels in diabetic rats after the intraportal injection of INS-lentiviral particles, highlighting a promising method for achieving long-term glycemic control [20]. Similarly, Saaristo et al. reported enhanced angiogenesis and wound healing in diabetic mice using an adenoviral vector expressing VEGF-C, which could translate into the better management of diabetic ulcers and ischemic conditions [21]. Callejas et al. extended these findings to diabetic canines and mice, showing a long-term improvement in glucose homeostasis and insulin production with the intramuscular delivery of AAV vectors expressing insulin and glucokinase [11]. Moreover, Sapir et al. demonstrated enhanced blood glucose control and insulin sensitivity in diabetic mice using AAV delivery of INS, PDX1, and GCK, further supporting the efficacy of gene therapy in regulating metabolic functions [22]. These animal studies collectively validate the potential of gene therapy as a transformative approach for diabetes treatment, providing a robust preclinical basis for advancing to human trials and ultimately improving clinical outcomes for diabetes patients.

### 4.2. General Interpretation of Human Studies

The current human studies on gene therapy for diabetes present significant implications for the future of diabetes treatment and management. The study by Ropper et al. showed that plasmid VEGF can effectively alleviate neuropathic symptoms and reduce pain in patients with type 1 and type 2 diabetes, demonstrating the potential for gene therapy to address diabetic complications beyond glycemic control [27]. Hsu, P. Y. J. et al. highlighted long-term improvements in glucose and insulin production using recombinant adeno-associated virus (rAAV) to carry the human insulin gene, indicating the possibility of achieving sustained glycemic control in diabetic patients [24]. Kupczynska et al. demonstrated an improved healing of ischemic lesions and increased angiogenesis in diabetic foot syndrome patients using a bicistronic VEGF165/HGF plasmid, which could lead to the better management of diabetic wounds and the prevention of limb amputation [28]. Kessler et al. reported significant pain reduction and potential disease-modifying effects in patients with painful diabetic peripheral neuropathy treated with VM202, a gene therapy involving hepatocyte growth factor (HGF), suggesting a novel therapeutic option for this challenging condition [26]. Additionally, Barc et al. showed that the intramuscular injection of pIRES/VEGF165/HGF in type 2 diabetes patients with critical limb ischemia resulted in increased serum VEGF levels, improved ankle–brachial index (ABI), decreased rest pain, and enhanced vascularization, offering hope for patients with severe vascular complications [25].

### 4.3. Limitations

In this review, we collated studies that showed the ingenuity and effectiveness of gene therapy in human and animal models. It is important to distinguish the limitations of animal studies compared to human trials; studies pertaining to the success of gene therapy in the treatment and management of diabetes performed on animals have limitations. Using suggestions from each type of study, we can attempt to bridge the gap between the preclinical successes and, consequently, human applications. The differences between the studies would include metabolic processes, immune responses, and the overall complexity of diabetes mellitus in humans compared to animal models. Callejas’ study noted the difficulty of translating his findings due to the differences in immune responses between canines and humans. Another approach taken was that of Kojima, who induced islet neogenesis in the liver using NeuroD and Betacellulin to regenerate insulin-producing cells [16]. Handorf focused his study on type 1 diabetic mellitus and showcased the efficacy of gene therapy in improving glycemic control in diabetic mice [23]. Sapir used PDX-1 to transdifferentiate liver cells into insulin-producing cells, lowering the blood glucose in diabetic mice [22]. In light of this, studies are included which show the potential of being translated to the treatment of human diabetes mellitus.

Despite the promising findings, this review manuscript has several limitations that must be acknowledged. First, the variability in study designs, sample sizes, and follow-up durations among the included studies makes it challenging to draw definitive conclusions about the efficacy and safety of gene therapy for diabetes. Many studies, particularly those involving human participants, have small sample sizes and short follow-up periods which may not capture the long-term effects and potential adverse outcomes of these therapies. Additionally, the heterogeneity in delivery methods and target genes further complicates the ability to compare outcomes across studies directly. Another limitation is that the lack of standardized protocols and outcome measures across studies limits the generalizability of the results. Finally, while the included studies explore various aspects of gene therapy for diabetes, they do not comprehensively cover all possible gene targets and therapeutic approaches, leaving gaps in the current knowledge base. Addressing these limitations in future research will be crucial for advancing gene therapy as a viable treatment option for diabetes.

### 4.4. Implications

The current body of research into gene therapy for diabetes, encompassing both human and animal studies, provides a promising outlook for future therapeutic strategies. The human studies reviewed demonstrate potential clinical benefits, including improved glycemic control, enhanced insulin production, and significant pain reduction in patients with diabetic complications.

The implications of these findings are profound. If the positive outcomes observed in animal models and early human trials can be consistently replicated and scaled, gene therapy could revolutionize diabetes treatment. This approach offers the potential for long-term solutions, possibly reducing the need for ongoing pharmacological intervention and improving patients’ quality of life. Furthermore, advancements in delivery methods, such as the use of adeno-associated viruses (AAV) and bicistronic plasmids, enhance the precision and efficacy of these therapies, potentially minimizing side effects and improving patient outcomes.

## 5. Conclusions

### 5.1. Healthcare Providers’ Role in Gene Therapy

**Figure 2:**
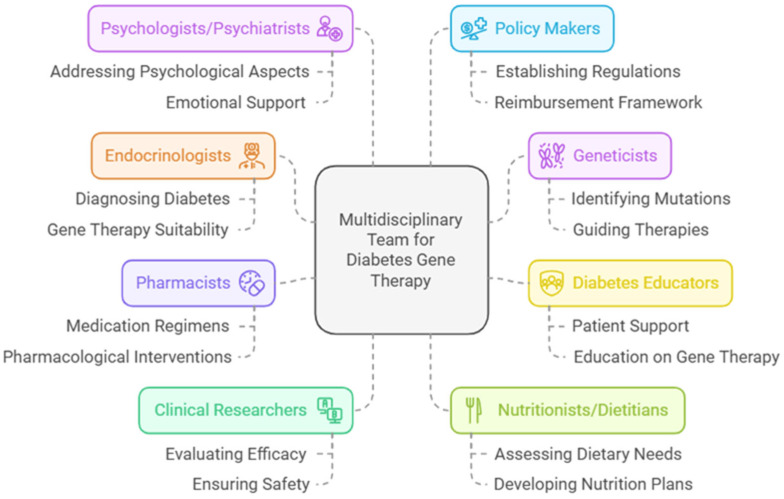
Sample of the multidisciplinary team role for diabetes gene therapy.

Gene therapy represents a groundbreaking advancement in the management of diabetes, with promising results in both glycemic control and the treatment of complications. While the direct application and administration of gene therapies predominantly lie within the scope of specialized medical professionals and researchers, the integration of these therapies into diabetes care requires the coordinated efforts of an interdisciplinary team. Endocrinologists can oversee the selection of appropriate candidates for gene therapy, monitor therapeutic efficacy, and manage potential adverse effects [29]. Nurses and diabetes educators can bridge the gap between advanced therapies and patient understanding, fostering informed decision-making. As for pharmacists’ role, although gene therapy is outside the direct scope of pharmacy practice, pharmacists play a crucial role in the broader framework of precision diabetes care. They ensure the compatibility of gene therapies with ongoing pharmacological regimens and counsel patients on managing any drug–gene interactions [30]. As gene therapy emerges as a potential treatment modality for diabetes, integrating pharmacogenomic principles will be essential to optimize therapeutic efficacy and safety [31]. Figure 2 illustrates the multidisciplinary roles of healthcare providers in the implementation and management of gene therapy for diabetes, highlighting the interconnected responsibilities of clinical pharmacists, physicians, genetic counselors, and allied health professionals in ensuring comprehensive patient care and successful therapeutic outcomes.

### 5.2. Future Trends

As research techniques advance, scientists continue to make new findings and advancements. The most promising success to date is the use of gene therapy in the treatment of sickle cell disease. Gene addition or silencing has been shown to be very successful in producing new healthy hemoglobin [32]. The world’s first clinical trial to test gene therapy for T1D is currently recruiting patients to transplant genetically engineered pancreatic islet cells in Australia [33]. This first-in-human safety and efficacy trial is aimed at type 1 diabetes (T1D) patients and explores the potential of genetically engineered islet cells delivered via a recombinant adeno-associated virus (AAV) vector. The trial seeks to restore glucose control by promoting insulin production, a significant innovation in diabetes treatment.

As gene therapy for diabetes continues to evolve, future trends will likely center around refining and implementing robust evaluation matrices to assess the long-term impacts, patient outcomes, and cost-effectiveness of these innovative treatments. One key area of focus will be long-term safety regarding the integration of genetic material by viral vectors. [34]. In addition to safety, patient outcomes will be a critical measure, encompassing not only improvements in glycemic control, but also factors such as quality of life, symptom relief (particularly in neuropathy and wound healing), and functional status. Addressing cultural and social barriers will also be crucial in ensuring equitable access to gene therapy [35]. Factors such as health literacy, cultural beliefs, socioeconomic challenges, and systemic healthcare disparities must be considered to foster acceptance and ensure inclusiveness in treatment delivery. It is imperative that healthcare providers from different domains adapt to the growing scope of gene therapy and gain expertise in the new therapeutic areas for this metabolic disease. Beyond diabetes, gene therapy will likely expand to treat a wider range of conditions, including genetic disorders, cancers, and neurodegenerative diseases.

## Figures and Tables

**Table 1:** Summary of selective gene therapy studies.

Study	Study Population	Design	Follow-Up	Sample Size	Delivery Method	Long-Term Efficacy	Target Genes
**Type 1 and Type 2 Diabetes Participants**							
Ropper et al. [27]	T1D, T2D	Controlled trial	6 months	50	Plasmid VEGF	Improvement in diabetic neuropathic symptoms and pain reduction	VEGF
Hsu, P. Y. J. et al. [24]	T1D, T2D	Controlled trial	5 years	N/A	Recombinant adeno-associated virus (rAAV)	Glucose and insulin production, glycemic control	Human insulin gene driven by rat insulin promoter
**Complications from Diabetes Mellitus**							
Kupczynska et al. [28]	Diabetic foot syndrome patients	Randomized controlled trial	6 months	N/A	Intramuscular injection of bicistronic VEGF165/HGF plasmid	Improved healing of ischemic lesions and increased angiogenesis	VEGF165, HGF
Kessler et al. [26]	Painful diabetic peripheral neuropathy patients	Phase III randomized controlled trial	9 months (DPN 3-1), 12 months (DPN 3-1b)	N/A	Intramuscular injection of VM202	Significant pain reduction in DPN 3-1b, potential disease-modifying effects	HGF
Barc et al. [25]	Type 2 diabetes patients with critical limb ischemia	Randomized controlled trial	3 months	28	Intramuscular injection of pIRES/VEGF165/HGF; 4 mg of bicistronic plasmid	Statistically significant increase in serum VEGF levels, improvement in ankle–brachial index (ABI), decreased rest pain, improved vascularization in CTA	VEGF125, HGF
**Type 1 Diabetes Participants Only**							
Handorf, M. et al. [23]	Type 1 diabetic rats	Randomized controlled trial	18 months	N/A	AAV	Sustained insulin production as well as glycemic control	INS
**Animal Models Only**							
Kojima, H. et al. [16]	Diabetic mice	Animal study	N/A	N/A	Lentivirus	Improved islet neogenesis as well as insulin production	NeuroD1, Betacellulin
Elsner, M. et al. [20]	Diabetic rats	Animal study	12 months	N/A	Intraportal injection of INS-lentiviral particles	Stable hepatic insulin expression and blood glucose levels, reversal of diabetes	INS
Saaristo et al. [21]	Diabetic mice	Controlled trial	35 days	N/A	Adenoviral vector expressing VEGF-C	Enhanced angiogenesis and wound healing	VEGF-C
Callejas et al. [11]	Diabetic canines and mice	Clinical trial	4 years	N/A	Intramuscular delivery of AAV vectors expressing insulin and glucokinase	Improvement in long-term survival, glucose homeostasis, and insulin production	INS, GCK
Sapir et al. [22]	Diabetic mice	Controlled trial, animal and human participants	N/A	N/A	AAV	Improvement in blood glucose control as well as insulin sensitivity	INS, PDX1, GCK

Abbreviations: ABI: ankle–brachial index; AAV: adeno-associated virus; CLI: critical limb ischemia; FOXP3: forkhead box P3; GCK: glucokinase; GLP1: glucagon-like peptide 1; HGF: hepatocyte growth factor, INS: insulin gene; NeuroD1: neurogenic differentiation 1; PDX1: pancreatic and duodenal homeobox 1; VEGF: vascular endothelial growth factor; VEGF165: vascular endothelial growth factor 165 isoform; VEGF-C: vascular endothelial growth factor-C; VM202: non-viral gene therapy expressing two isoforms of hepatocyte growth factor.

## Data Availability

This narrative review is based on publicly available information, including peer-reviewed articles, guidelines, and other published resources. As this study does not involve original data generation, no primary datasets were created or analyzed. The supporting materials and references cited in the review are available in the public domain and can be accessed through the referenced journals or publishers.

## Abbreviations

ABI	Ankle–brachial index
AAV	Adeno-associated virus
CLI	Critical limb ischemia
DPN	Diabetic peripheral neuropathy
FOXP3	Forkhead box P3
GLP1	Glucagon-like peptide 1
GCK	Glucokinase
HGF	Hepatocyte growth factor
INS	Insulin gene
PDX1	Pancreatic and duodenal homeobox 1
VEGF	Vascular endothelial growth factor
VEGF-C	Vascular endothelial growth factor-C
VEGF165	Vascular endothelial growth factor 165 isoform
VM202	Non-viral gene therapy expressing two isoforms of hepatocyte growth factor

## Appendix A

**Table A1:** Risk of bias assessment MMAT.

Study	Study Type	Clear Research Questions	Does the Collected Data Allow Us to Address the Research Questions?	Criteria 1	Criteria 2	Criteria 3	Criteria 4	Criteria 5
Risk of Bias Assessment Using 2018 MMAT Checklist for Type 1 and Type 2 Diabetic Human Participants								
**Ropper et al.** [27]	Randomized controlled trial	Yes	Yes	Yes, the study used a double-blind, placebo-controlled design with a vigorous randomization protocol	Yes, the baseline characteristics were similar between the groups	Yes, the study had reported primary outcome data for all patients	Yes, the researchers were also blinded	Yes, the participants were diligent in their groups for the study period
**Hsu, P. Y. J. et al.** [24]	Randomized controlled trial	Yes	Yes	Yes, the participants were adequately randomized using computer-generated algorithms	Yes, the study had intervention and control groups with similar baseline characteristics	Yes, the study was able to compile all the data for all participants	Yes, the researchers were also blinded	Adherence was over 70%, there were several participants who did not complete the study
**Kupczynska et al.** [28]	Randomized controlled trial	Yes	Yes	Yes, sealed envelopes were used for randomization	Yes, the baseline characteristics were comparable for the groups	Yes, the complete outcome data were reported	Yes, the researchers were blinded to the intervention	Strong adherence and maintenance of intervention in the assigned groups
**Kessler et al.** [26]	Randomized controlled trial	Yes	Yes	Computer-generated algorithm randomization	Yes, the baseline characteristics were similar between groups	Yes, the complete outcomes were reported	Yes, the researchers were blinded	There was very high adherence to the intervention in the control group
**Barc et al.** [25]	Randomized controlled trial	Yes	Yes	Block randomization	Yes, the groups are similar in baseline characteristics	Yes, the complete outcome data were provided	Yes, the researchers were blinded in the study	There were several participants who did not complete the study

**Table A2:** A Risk of Assessment using CONSORT tool.

Risk of Bias Assessment for Animal Studies Using Consort Outcomes 2022 Checklist						
Criteria	Kojima, H. et al. [16]	Elsner, M. et al. [20]	Handorf, H. et al. [23]	Saaristo, A. et al. [21]	Callejas, D. et al. [11]	Sapir, T. et al. [22]
**Title and Abstract**	“NeuroD-Betacellulin Gene Therapy Induces Islet Neogenesis in the Liver and Reverses Diabetes in Mice”—clearly represents the study by mentioning a gene therapy technique and its use in animal models.	“Reversal of Diabetes Through Gene Therapy of Diabetic Rats by Hepatic Insulin Expression Via Lentiviral Transduction”—clear title and abstract about the use of gene therapy in animal models.	“Insulin Gene Therapy for Type 1 Diabetes Mellitus”—mentions both the target and method to be utilized. The abstract summarizes the study exceptionally.	“Vascular Endothelial Growth Factor-C Accelerates Diabetic Wound Healing”—mentions the role of VEGF-C and the specific group.	“Treatment of Diabetes and Long-Term Survival After Insulin and Glucokinase Gene Therapy”—indicates the focus on treatment of gene therapy.	“Cell-replacement therapy for diabetes: Generating functional insulin-producing tissue from adult human liver cells”—represents a method of cell replacement therapy, and the abstract mentions the induction of insulin-producing cells from liver cells via PDX-1 with the potential to ameliorate hyperglycemia in diabetic populations.
**Background and Objectives**	NeuroD and Betacellulin were used to induce islet neogenesis and reverse diabetes, mentioning prior research has shown that these genes can stimulate islet cell growth and function.	Using lentiviral vectors to induce insulin production in liver cells, essentially creating a new source of insulin for diabetic rats.	This study focuses on the potential treatment options of insulin gene therapy rather than the conventional methods used for type 1 diabetic populations due to the limitations of the current insulin therapy.	Reasoning behind the use of VEGF-C and its potential for angiogenesis and lymphangiogenesis	Detailed reasoning behind using insulin and glucokinase gene therapy to make a glucose sensor in skeletal muscle. Also addresses the limitations of exogenous insulin therapy.	Using pancreatic and duodenal homeobox gene 1, also known as PDX-1, to induce liver cells to produce insulin. The objective is to determine the efficacy of PDX-1-induced transdifferentiation of adult human liver cells into functional insulin-producing cells as a potential autologous cell replacement therapy option for diabetes patients.
**Methods**	Constructed viral vectors capable of carrying NeuroD and Betacellulin genes, using streptozotocin administered via tail vein injection.	Constructed lentiviral vectors to express insulin, using streptozotocin. Administered via hepatic portal vein injection.	Illustrates their use of viral vectors to target specific cells, and the construction of these viral vectors carrying the human insulin gene. Transducing hepatocytes in diabetic mice by initiating multiple low doses of streptozotocin to destroy pancreatic beta cells.	Adenoviral vectors encoding VEGF-C, VEGF-C156S, VEGF-A165, VEGF-B186, and control vectors construction. Injected intradermally around full-thickness punch biopsy wounds in diabetic mice.	Adeno-associated viral vectors carrying human insulin and glucokinase genes were produced by triple transfection of HEK 293 cells. Purification process using CsCl-based gradient method. Diabetes induced in male Beagle dogs using streptozotocin, 35 mg/kg, and alloxan, 40 mg/kg. Delivered intramuscularly to multiple sites on the dogs’ hind legs, with doses of 1,000,000,000,000 vg/kg or 2,000,000,000,000 vg/kg.	Isolated adult human liver cells from liver transplantation surgeries and fetal human livers from deliberate abortions. Then, hepatocytes were cultured and infected with recombinant adenoviruses encoding PDX-1 under the control of the cytomegalovirus promoter. Also, soluble factors, such as EGF and nicotinamide, were used as a way to enhance transdifferentiation.
**Participants**	C57BL/6 mice, 8 to 10 weeks (about 2-and-a-half months) old, who had diabetes mellitus induced through multiple low doses of streptozotocin.	Wistar rats, 10 to 12 weeks (about 3 months) old, diabetes mellitus induced through a single high dose of streptozotocin.	Male C57BL/6 mice; 8–10 weeks old.	10-week-old obese diabetic mice (BKS.Cg-m+/+ Lepr db/db) and 8–10-week-old C57BLKS mice.	6–12-month-old male beagle dogs. Diabetes induced by a single intravenous injection of streptozotocin and alloxan.	Used adult and fetal human liver tissue; adult liver cells obtained from eight liver transplantation surgeries from children aged 4–10 years old and three participants over 40 years old, fetal human tissues were used from four deliberate abortions at 20–22 weeks of gestation.
**Interventions**	A single injection of a viral vector comprising NeuroD and Betacellulin genes, while the control group received a saline injection.	A single injection of a lentiviral vector which expresses insulin in the liver, while the control group received a saline injection.	A single injection of a constructed viral vector carrying the human insulin gene via tail vein injection.	Intradermal injections of adenoviral vectors encoding VEGF-C, VEGF-C156S, VEGF-A165, VEGF-B186, VEGFR-2-lg, VEGFR-3-lg, or LacZ (control) around the wound. Then, they were covered with a sterile transparent occlusive dressing and monitored for wound closure and tissue repair.	Intramuscular injections of AAV vectors encoding human insulin and glucokinase genes. The control group included dogs with exogenous insulin and dogs receiving only one of the genes.	Treating hepatocytes with adenoviruses encoding PDX-1 and soluble factors to induce transdifferentiation into insulin-producing cells. The control group included untreated hepatocytes as well as cells treated with adenoviruses lacking the PDX-1 gene.
**Outcomes**	Primary outcomes: Blood glucose levels and serum insulin levels. Secondary Outcomes: Histological analysis of liver and pancreatic tissues to assess islet neogenesis.	Primary outcomes: Blood glucose levels and serum insulin levels. Secondary Outcomes: Histological examination of liver tissue to assess insulin expression.	Primary Outcomes: Fasting blood glucose levels, serum insulin levels. Secondary Outcomes: Body weight, serum albumin levels, liver histology, expression of the insulin gene in liver tissues.	Primary Outcomes: Wound closure rates, angiogenesis, lymphangiogenesis. Secondary Outcomes: Recruitment of inflammatory cells and expression of VEGF-C in wound tissues.	Primary Outcomes: Fasting blood glucose levels, serum insulin levels, glucokinase activity. Secondary Outcomes: Body weight, serum fructosamine levels, oral glucose tolerance test (OGTT) results, absence of secondary complications, such as cataracts.	Primary Outcomes: Expression of insulin and other pancreatic genes, insulin content, secretion in response to glucose. Secondary Outcomes: Ability of transdifferentiated cells to ameliorate hyperglycemia in diabetic mice and long-term functionality in vivo.
**Sample Size**	10–12 mice per group, 20 total mice.	15 rats per group, 30 total rats.	20 mice were used; 10 mice in the treatment group and 10 mice in the control group.	Four to six mice with paired wounds.	10 dogs were used in total in various groups.	Liver tissue from different donors in vitro experiments, 15 diabetic NOD-SCID mice for in vivo transplantation studies.
**Randomization**	Computer-generated random sequence divided the mice into intervention vs. control groups	Random number generator used to divide the rats into intervention vs. control groups	Randomized through a computer-generated random sequence	Random assignment of the mice was performed to different treatment groups	Random assignment to treatment or control groups	Random assignment to receive either transdifferentiated adult human liver cells or control untreated adult human liver cells
**Blinding**	Blinding of the outcome was not specified; therefore, bias in the measurement of outcomes is a factor to consider	Blinding of the researchers were not mentioned	Not available	Not available	Not available	Not available
**Statistical Methods**	ANOVA which compares blood glucose and insulin levels between groups; *p*-value < 0.05.	*t*-tests for comparing the blood glucose and insulin levels; *p*-value < 0.05.	ANOVA for comparing blood glucose and serum insulin levels, *T*-tests for pairwise comparisons.	Two-tailed Student’s *t*-tests for comparing differences between groups.	Two-tailed Student’s *t*-tests for comparing differences between groups	Two-tailed Student’s *t*-tests for comparing differences between groups
**Results**	The intervention group had shown a drastic reduction in blood glucose levels of about 50%. The intervention group also had a serum insulin increase compared to almost zero for the control group. With histological analysis, study confirmed increased islet neogenesis of the liver.	The intervention group showed a significant reduction in blood glucose levels, about 60%, as well as increased serum insulin levels compared to the control group. Histological analysis did confirm greater insulin expression in the liver.	Treatment group showed a significant reduction in their fasting glucose levels, with an average reduction of 50%, as well as an increase in serum insulin levels compared to the control group.	VEGF-C-treated wounds show significantly accelerated wound closure than the control group. The treatment group had a 20% reduction in wound size in 9 days and wound closure was complete, on average, in 21 days, compared to 26 days for the control group.	Treatment group had significant improvement in glycemic control and fasting blood glucose levels. Fasting blood glucose levels were normalized within 1–2 weeks after gene therapy, and were considered stable for over 4 years.	Significant activation of pancreatic genes and insulin production in the treatment group. A sevenfold increase in insulin gene expression compared to control group.
**Participant Flow**	10 mice in each group completing the study	15 rats in each group completing the study	Mentioned in study, no mice were lost to follow-up	All mice completed the study with data collected at 6 days, 18 days, and date of wound closure	All dogs completed study with a follow-up of 4 years	No samples of AHL cells or mice were lost to follow-up and all 15 mice completed the in vivo study
**Recruitment**	Recruitment was the acquisition of lab mice without diabetes, induction of diabetes followed after	Recruitment was the acquisition of lab rats without diabetes, induction of diabetes followed after	Over a two-week period	Induction of diabetes and creation of punch biopsy wounds were conducted to recruit the mice	Included induction of diabetes and administration of gene therapy	Included collection of liver tissue samples
**Baseline Data**	Age, weight, initial blood glucose levels were recorded and similar in both groups	Age, weight, initial blood glucose levels were recorded and similar in both groups.	Age, weight and initial blood glucose levels were similar in the two groups	Age and weight were similar in the treatment and control groups	Age, weight, initial blood glucose levels were similar in the groups	Donor age, tissue source, and initial cell viability were similar across the groups.
**Numbers Analyzed**	Every participating animal completed the study	Every participating animal completed the study	All 20 mice were included in the final analysis	All mice in each group were included in final analysis	All 10 dogs were included in the final analysis	All samples and mice included in final analysis
**Outcomes and Estimations**	Blood glucose levels and insulin levels	Blood glucose levels and insulin levels	Demonstrated a significant reduction in blood glucose levels and higher insulin levels in the treatment group	Significantly faster wound closure and enhanced angiogenesis and lymphangiogenesis in VEGF-C-treated wounds	Showed significantly improved glycemic control, insulin levels, and glucokinase activity in treatment group	Significant increase in insulin production, glucose-regulated insulin secretion, and activation of pancreatic genes
**Ancillary Analyses**	Gene expression study in hepatic cells, to confirm expression of NeuroD and Betacellulin	Liver function testing to ensure hepatic insulin expression	Gene expression studies in hepatocytes confirm the expression of the insulin gene. As well as analyses of serum albumin levels and liver function	Immunohistochemical staining for VEGF-C expression, quantification of blood and lymphatic vessels, and recruitment of inflammatory cells. Soluble receptor inhibitors were used to conduct analysis on the role of endogenous VEGF-C/VEGF-D	Detailed histological examination of muscles and pancreas tissues, confirmation of gene expression and enzyme activity	Immunofluorescence and electron microscopy were used to confirm the insulin production and storage, quantitative RT-PCR for gene expression analysis, as well as glucose tolerance tests
**Harms**	No significant adverse effects	No significant adverse effects	No significant adverse effects were reported	No significant adverse effects were reported	No significant harm or adverse effects were reported	No significant harm or adverse effects were reported
**Discussion**	Focuses on the potential of incorporating these findings into human studies	Focuses on the implications for human gene therapy	The potential to translate these results to human participants is emphasized, as well as the implications for long-term diabetes management	The discussion highlights the potential for translating these results to humans for diabetes management, and emphasizes the success of VEGF-C in enhancing the angiogenesis and lymphangiogenesis to accelerate the wound healing process	Highlights the potential to translate these results into human participants with diabetes and achieve long-term glycemic control, as well as preventing diabetes complications	Highlights the potential to replicate these results in human participants. Emphasizes the success of PDX-1-induced transdifferentiation of liver cells into insulin-producing cells. Authors also discuss the implications of this therapy for diabetes management and the potential to reduce or eliminate the need for donor islets and immunosuppression
**Limitations**	Did not include a long-term follow-up	Potential to duplicate results in human studies	The results of animal models are potentially applicable to humans, follow-up lasted only 12 weeks	More research is needed to assess long-term effects and optimize delivery methods	Dog model, which may not be able to translate into human population	Limitations of islet transplantation, donor shortages and the need for immunosuppression
**Generalizability**	Further research would be needed to discuss generalizability to human studies	Further research would be needed to discuss generalizability to human studies	Further research would be needed to confirm the efficacy and safety in humans	Authors suggest the utilization of VEGF-C to be a valuable therapeutic tool for enhancing wound healing	Authors suggest that further similar approaches to gene therapy in diabetic patients be conducted	Further research and clinical trials necessary to confirm efficacy and safety in humans, as well as to optimize the process for diverse patient populations
**Interpretation**	Highlights novel approach of islet neogenesis through gene therapy	Highlights the potential of hepatic insulin gene therapy	Highlights the approach of insulin gene therapy and its potential effectiveness in diabetic populations	Highlights the novel approach of VEGF-C in the improvement of wound healing and its improved outcomes in diabetic patients	Highlights the approach of insulin and glucokinase gene therapy, with its potential to revolutionize treatment for diabetic patients	Highlights the approach of using PDX-1 and SFs to induce liver-to-pancreas transdifferentiation and its potential for treatment in human diabetic patients
**Other Information**	-	-	-	-	-	-
**Registration**	Not needed for animal study	Not needed for animal study	Not applicable	Not applicable	Not applicable	Not applicable
**Protocol**	Detailed protocol is available	Detailed protocol is available upon request and granted permission	Detailed protocol is available	Detailed protocol is available	Detailed protocol available	Detailed protocol available
**Funding**	Grants awarded from the National Institutes of Health and private foundations	Grants awarded from academic institutions and private research funds	Funded by grants from the National Institutes of Health and other private foundations	Funded by the Academy of Finland, the European Union, the National Institutes of Health, the Novo Nordisk Foundation, the Finnish Diabetes Foundation, the Maud Kuistila Foundation, and the Finnish Cultural Foundation	Funds granted from the Ministerio de Ciencia e Innovacion, Generalitat de Catalynya, the European Commission, the National Institutes of Health, the Howard Hughes Medical Institute	Funded by the Juvenile Diabetes Research Foundation, the Israel Science Foundation

Abbreviations: Vg/kg = viral genomes per kilogram of body weight; ADL = adult human liver, SF = soluble factors.
